# Interpersonal relationships and communication as a gateway to patient and public involvement and engagement

**DOI:** 10.1111/hex.12683

**Published:** 2018-03-25

**Authors:** Mary Chambers

**Affiliations:** ^1^ Faculty of Health Social Care and Education Kingston University and St. George’s, University of London Cranmer Terrace London SW17 ORE

Internationally, health‐care systems and organizations are constantly endeavouring to improve performance, enhance patient and carer experience and achieve better patient outcomes. To facilitate this, health and social care professionals are increasingly involving patients and the public in a range of decision‐making processes, person‐centred care planning,^1^, ^2^ development of clinical guidelines and care delivery systems, research strategies and protocols and organizational and government policies.

Engagement with patients and carers, no matter whether specific or general, requires good communication skills and trusting interpersonal relationships. These core skills apply not only to the patient‐practitioner relationship, but also to interprofessional relationships and team communications. Increasing patient involvement (PI) in care decisions and greater partnerships between patients and health‐care professionals (HCPs) will help ensure improved patient safety and enhanced patient satisfaction. To facilitate this, guidelines^3^ and frameworks can be helpful, whilst acknowledging and respecting the need for flexibility depending on the care context.

The papers included in this issue of HEX highlight the variety of ways in which patients and public can, and do, contribute to service and policy development, influence the nature and conduct of research and participate in decision making from individual care planning to guidelines and frameworks. Highlighted in most papers is the importance of interpersonal relationships and good communication skills.

Patient and public involvement (PPI) in both system and organization development is described in the papers of Hindi et al*,* Souliotis et al*,* Perez et al and Russell et al*,* each employs a different approach, demonstrating the importance of mapping the method of enquiry to purpose. Hindi et al conducted a United Kingdom (UK)‐based systematic review of evidence on patient and public perspectives regarding existing community pharmacy services, extended pharmacist roles and strategies used to raise awareness of pharmacy services. The authors present a detailed account of factors facilitating the use of pharmacy services and how physicians can influence public perception of pharmacy services.

When wanting to engage the public regarding opinions on excessive use of unwarranted medical care, Perez et al employed a civic engagement approach based on social values. Unwarranted medical care is a concern for health‐care policymakers, but strategies to reduce overuse may threaten aspects of health‐care delivery valued by the public. In groups, and using case scenarios, the public were asked to address the problem of medicines overuse and requested to choose the most acceptable reduction strategy. Most of the perspectives offered were congruent with those already being initiated or discussed at provider level. Engaging the public in the decision‐making process creates a sense of ownership increasing the likelihood of successful implementation.

Souliotis et al, working with a European‐wide cancer patient organization (CPO), choose an online self‐report questionnaire when exploring the degree and impact of CPO participation in health policy. As a previously understudied area, with a small number of mainly country‐specific qualitative studies suggesting that, despite the growing number of patient organizations in Europe and their increasing involvement in policy issues, political influence was limited. Souliotis et al obtained similar results, reinforcing the position that a higher degree of patient participation does not necessarily translate into greater impact, although interlinked.

The paper by Russell et al forms a link between community engagement and PPI in research. Again, with a European focus and using case study methodology, this study focussed on the development of drugs to treat autism. A consortium, funded to explore the underpinning biological mechanisms of autism, used the knowledge generated to develop effective pharmacological and other interventions to treat autism. A promotional video, of interviews with scientists working on biomedical studies as part of the consortium, was used at two PPI events. The video acted as a prompt to solicit comments about the consortium's project agenda. Data, in the form of open comments, were collected either during or after these events. This study highlights that “*selective PPI*” is not advisable.

When wanting to increase patients’ awareness of research and encourage recruitment to projects, organizations can employ a variety of approaches. Using case study methodology, Wienroth et al describe how one clinical department piloted an initiative where a research statement was inserted in letters requesting patients to attend an outpatient appointment for the first time. Considerable thought was given to the wording of the statement by both staff and lay members with extensive PPI experience in research. Using a pre‐post intervention survey, the findings suggest that, despite the attention given to the construction of the statement, only a tiny minority found it *very clear*. The statement was not thought to explain the concept of research and of little help in encouraging research participation. Findings indicate that a simple, single solution is not the answer to either raising patients’ awareness of research or increasing patient‐initiated recruitment.

The case study reported by Brown et al relates to openness, inclusion and transparency in the practice of public involvement (PI) in research and how reflection can increase understanding. The authors held a reflective exercise and solution‐focused workshop to explore perceptions of how an approach to PI grounded in the principles of openness, inclusion and transparency was experienced and how it might be used to improve PI practice. The findings indicated practical issues around roles and responsibilities, the use of language and information overload when working with community representatives.

A number of manuscripts in this issue consider patient‐centred care (PCC). Santana et al offer a conceptual framework on how to practice PCC. Following a literature review, and using the Donabedian model for health‐care improvement, a generic conceptual framework was developed in collaboration with patient and family caregiver representatives. This framework potentially offers a “*stepwise roadmap to guide health‐care systems and organizations in the provision PCC.”*

From an organizational perspective, Horrell et al. outline the development of the Person Centred Coordinated Care Organisational Change Tool (P3C‐OCT). Development began with a literature review, a scoping review of National Health Service (NHS) guidelines and scrutiny of policy documents; development of domains, subdomains and component activities followed. There were extensive stakeholder engagement and validation through codesign. The tool based on the principles of promoting PCC is the first of its kind with potential for use across the NHS.

Multimorbidity is the theme of the papers by Vermunt et al and Knowles et al. For Vermunt et al, their objective was to examine the concept of goal orientation from the clinician's perspective, in the context of collaborative goal setting (CGS) and shared decision‐making (SDM), with patients experiencing multiple long‐term conditions. The findings suggest three types of goals; disease‐specific, functional and fundamental goals and authors conclude that the three‐goal model could facilitate collaborative goal setting in clinical practice but further research is needed.

Knowles et al emphasize that multimorbidity presents challenges for patients regarding safety and quality of care; hence, services redesign should be informed by patients and carer experience. The authors attempted to generate novel interventions with PPI to address safety in primary care and assess if participatory approaches were appropriate. Experience‐based codesign with a “trigger film to stimulate discussion” is described. The findings demonstrate that patients and professionals share a vision for improving primary care for patients with multimorbidity.

Technology can enhance communication, an essential aspect of PCC. The work of Belyeu et al, in which a mixed method study where patients with diabetes were given access to their doctors’ notes and After‐Visit Summaries. Before the study participants were generally positive about such access, although some worried about privacy and disruption to relationships. Two years later, both positive views and concerns remained. Authors concluded that electronic patient portals create both challenges and opportunities for patients, but stakeholders should be mindful that implementing such technology could add to health inequalities.

The manuscripts in this issue of HEX consider the importance of good interpersonal relationship and communication in health and social care, in patients with complex problems such as multimorbidity, and the potential contribution of technology to improved communication.
